# Clinical Features of Spontaneous Regression of Retinopathy of Prematurity in China: A 5-Year Retrospective Case Series

**DOI:** 10.3389/fmed.2021.731421

**Published:** 2021-08-31

**Authors:** Liang Wang, Manhong Li, Jun Zhu, Hongxiang Yan, Lei Wu, Jing Fan, Yi Zhou, Kaili Gou, Zifeng Zhang, Yusheng Wang

**Affiliations:** ^1^Department of Ophthalmology, Eye Institute of Chinese PLA, Xijing Hospital, Fourth Military Medical University, Xi'an, China; ^2^State Key Laboratory of Cancer Biology, Institute of Digestive Diseases, Xijing Hospital, Fourth Military Medical University, Xi'an, China

**Keywords:** fundus appearance, infants, regression, retinopathy of prematurity, cox proportional hazard regression model

## Abstract

**Purpose:** The aim of this study is to explore the clinical features of spontaneous regression of retinopathy of prematurity (ROP) in China, including fundus appearance, time course, and affecting factors.

**Methods:** Data of pediatric patients in whom ROP spontaneously regressed without treatment were collected, including general demographics, medical history, zones and stages of ROP, and changes of fundus appearance. The fundus manifestations of spontaneous regression in ROP were systematically summarized. Meanwhile, the time course of spontaneous regression in ROP was further analyzed, including the onset time, completion time, and duration of regression, which were all compared across different ROP zones and stages. The associated factors were analyzed by survival analysis for their correlation with delayed regression for the first time.

**Results:** Two hundred thirty-seven eyes of 237 pediatric patients were included. The fundus manifestations of regression differed across stages. Lesions gradually subsided, and the retinal vessels gradually vascularized completely. However, despite ROP regression, some abnormalities remained. We observed avascular retina in the temporal periphery (19.0%), increased vascular branching (6.8%), retinal pigmentary changes (6.8%), and smaller angle between the upper and lower temporal retinal vessel trunks (3.0%). Acute ROP started to regress at a median 40 weeks of postmenstrual age (PMA) and completely regressed by median 49.0 weeks of PMA. The median duration for regression was 8.5 weeks. The zone II ROP and stage 3 ROP had a later time for onset and completion of regression, and longer duration. Anemia and retinal hemorrhage (RH) were identified as independent risk factors for delayed regression by survival analysis.

**Conclusions:** During spontaneous regression, the fundus appearance is diverse, and the retinal vessels gradually vascularized completely. The time course of regression differs depending on the ROP zone and stage. Anemia and RH are independent risk factors for delayed regression. Further research of the natural course of the regression of ROP is needed to help design effective screening and follow-up plans.

## Introduction

Retinopathy of prematurity (ROP) is a vasoproliferative retinal disorder frequently seen in infants with low gestational age (GA) and birth weight (BW). Nowadays, it has already been the leading cause of childhood blindness worldwide (1). It is becoming a significant cause of avoidable blindness, especially in developing countries (2, 3). It was estimated that, in 2010, 184,700 patients had ROP worldwide, with 20,000 of them having severe visual impairment (4). Prompt detection of ROP in high-risk infants is crucial for managing severe retinopathy (5). ROP screening is highly heterogeneous globally. In underdeveloped regions, such as Northwest China, the number of medical professionals is insufficient to meet the demands imposed by ROP (6). However, most ROP cases regress spontaneously without treatment, and there were few reports on the clinical characteristics of regression of ROP. A better understanding of the natural course of regression of ROP will support the development of efficient strategies for ROP screening and prevention. A comprehensive investigation of the characteristics of spontaneous regression of ROP is needed. Thus, this retrospective study aimed to explore the clinical features and factors associated with spontaneous regression of ROP in China.

## Materials and Methods

Data of pediatric patients in whom ROP spontaneously regressed without treatment were collected from January 1, 2016, to December 31, 2019, in the Department of Ophthalmology, Xijing Hospital. This study was approved by the Ethics Committee of Xijing Hospital (KY20202099-C-1) and followed the principles of the Declaration of Helsinki. The infants were screened according to the 2014 Chinese screening guidelines of ROP (7). ROP was classified according to International Classification of Retinopathy of Prematurity revised in 2005 (8). Fundus examination was conducted weekly, fortnightly, or monthly, depending on the severity of the retinal lesions, until the completion of regression or longer. All the patients underwent a thorough fundus examination by RetCam (Clarity Medical Systems, Pleasanton, CA, USA), conducted by the same experienced pediatric ophthalmologist. The infants who were diagnosed as ROP by RetCam examination must be verified by another senior professional pediatric ophthalmologist using indirect ophthalmoscope (Keeler Company, Windsor, UK). Fundus images were recorded using the RetCam system at each follow-up. Fundus appearance, including ROP zones and stages, ridge, “popcorns,” retinal hemorrhage (RH), tortuous and dilated retinal vessels, and avascular zone of the peripheral retina, were analyzed.

Since the severity of binocular ROP lesions was identical in 197 of the 237 patients (83.1%), and the binocular eyes could not be considered as independent cases, the eye with more severe ROP was selected for analysis in each patient. If the severity of lesions was identical in both eyes, one eye was randomly selected.

Patients with stage 1–3 ROP, who were followed up until completion of spontaneous regression, were included. Patients with any of the following conditions were excluded: (1) patients who received treatment for ROP; (2) patients in whom ROP had already started to regress before the first examination in our hospital; (3) patients who were not followed up until the completion of regression; and (4) patients with cataract, glaucoma, or other comorbid eye diseases.

According to the research of the Cryotherapy for Retinopathy of Prematurity Cooperative Group (9), meeting any one of the following criteria was enough to define the onset of regression: (1) a decrease of at least two sectors in the number of sectors of the highest stage of acute ROP in a given zone in two consecutive examinations; and (2) recorded completion of vascularization in all sectors of a zone that previously had acute ROP. Meeting any one of the following criteria was enough to define the completion of regression: (1) lesions had faded completely, and complete retinal vascularization (the vessels on the nasal side reached the ora serrata, and the temporal vasculature was separated from the ora serrata by no more than one optic papilla diameter); and (2) despite the presence of abnormal changes, such as increased vascular branching, retinal pigmentary changes, ROP had regressed to a steady state, and the retinal vessels had grown to zone III.

The time of onset of regression was defined as the average time of two consecutive examinations showing acute ROP, without initiation of regression at the first of those examinations, and with a definite onset of regression at the second examination. Similarly, the time for completion of regression was defined as the average time of two consecutive examinations showing ROP at the earlier examination (before regression completion) and a definite regression completion at the next examination.

General demographic data, including sex, GA, BW, singleton or multiple pregnancy, and eutocia or cesarean, were recorded at the first visit. In addition, history of mother's gestational diseases (hypertensive disorders of pregnancy and gestational diabetes mellitus) and the patient's medical history [premature rupture of membranes, placenta previa, placental abruption, asphyxia, apnea, neonatal respiratory distress syndrome (NRDS), pneumonia, anemia (hemoglobin < 110 g/L), blood transfusion, septicemia, hyperbilirubinemia (total bilirubin > 1.5 mg/dl), congenital heart disease, and continuous positive airway pressure (CPAP)] were also recorded in detail. Data were gathered from the hospital medical records of patients. If the patients coming from other hospitals did not carry their medical records when they visited our hospital, we would investigate their medical history *via* telephonic interviews. BW < 1,000 g, GA < 28 weeks, RH, and the abovementioned factors were analyzed for their correlation with delayed regression.

### Statistical Analysis

The baseline characteristics of the ROP infants were summarized using descriptive statistics. The onset, completion, and duration times of regression of different ROP zones were analyzed by Wilcoxon rank sum test. The time course in different stages of ROP was investigated using the Kruskal–Wallis test. Related factors for delayed regression were examined by the Kaplan–Meier curve, and the differences were assessed by log-rank test. Cox proportional hazard regression model was further used to perform univariate analysis for the factors related to delayed regression. Statistically significant factors in the univariate analysis were incorporated into the multivariate Cox proportional hazard regression model to investigate whether these were independent factors affecting delayed regression. All the statistical analyses were performed with R software (Version 3.6.3, The R Foundation for Statistical Computing, Vienna, Austria) (packages: ggplot2, ggpubr, survival, surviminer, and tidyverse). Two-sided *p* < 0.05 was considered statistically significant.

## Results

### Patients and Data

**Figure 1:**
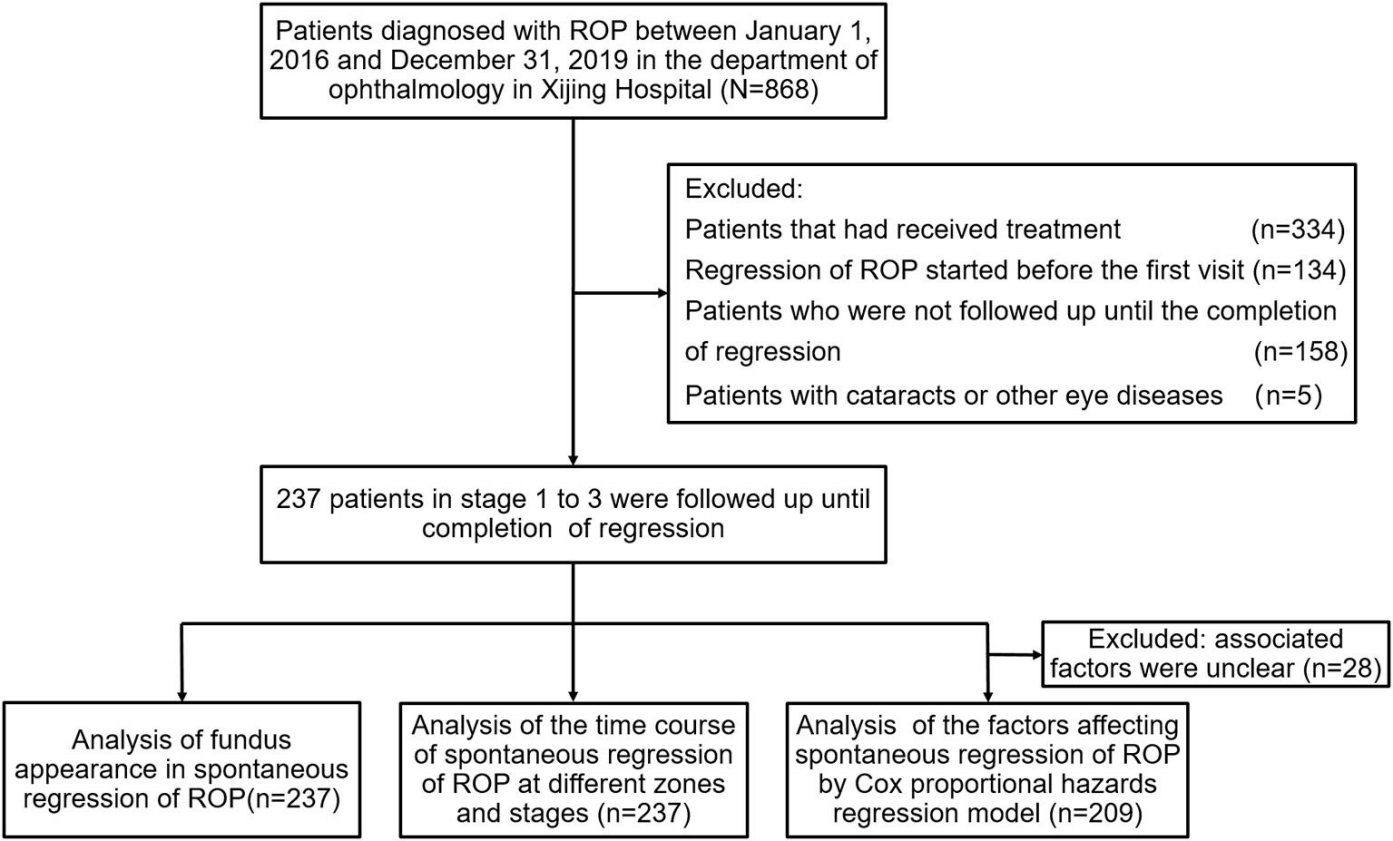
Flowchart of this study. ROP, retinopathy of prematurity.

The clinical data of 4,234 infants who had accepted fundus screening in the Ophthalmology Department of Xijing Hospital were collected, 868 of whom were diagnosed with ROP (20.5%). According to the inclusion and exclusion criteria of this study, 237 eyes of the 237 pediatric patients with ROP, which spontaneously regressed without treatment, were finally included. The flowchart of this study is shown in Figure 1. Table 1 summarizes their baseline characteristics. There were 125 males and 112 females. The mean GA of patients was 30.4 ± 2.15 weeks, and their mean BW was 1,418 ± 358 g. Mothers with multiple pregnancy accounted 22.3% of the cohort, and 59.1% patients were delivered by cesarean section; 210 patients (88.6%) presented bilateral ocular involvement, and 27 (11.4%) showed unilateral anomalies.

**Table 1:** Baseline characteristics of patients with regression of ROP.

**Parameters**	**Number**	**Percentage (%)**
**Sex**		
Male	125	52.7
Female	112	47.3
**Gestational age (GA)**		
<28 weeks	22	9.3
28–32 weeks	171	72.1
>32 weeks	44	18.6
**Birth weight (BW)**		
<1,000 g	22	9.3
1,000–2,000 g	204	86.1
>2,000 g	11	4.6
Multiple pregnancy	54	22.3
Caesarean	140	59.1
**Laterality**		
Unilateral ROP	27	11.4
Bilateral ROP	210	88.6
**Zone**		
II	30	12.7
III	207	87.3
**Stage**		
1	35	14.8
2	188	79.3
3	14	5.9

*ROP, retinopathy of prematurity*.

### Fundus Appearance of Regression

**Figure 2:**
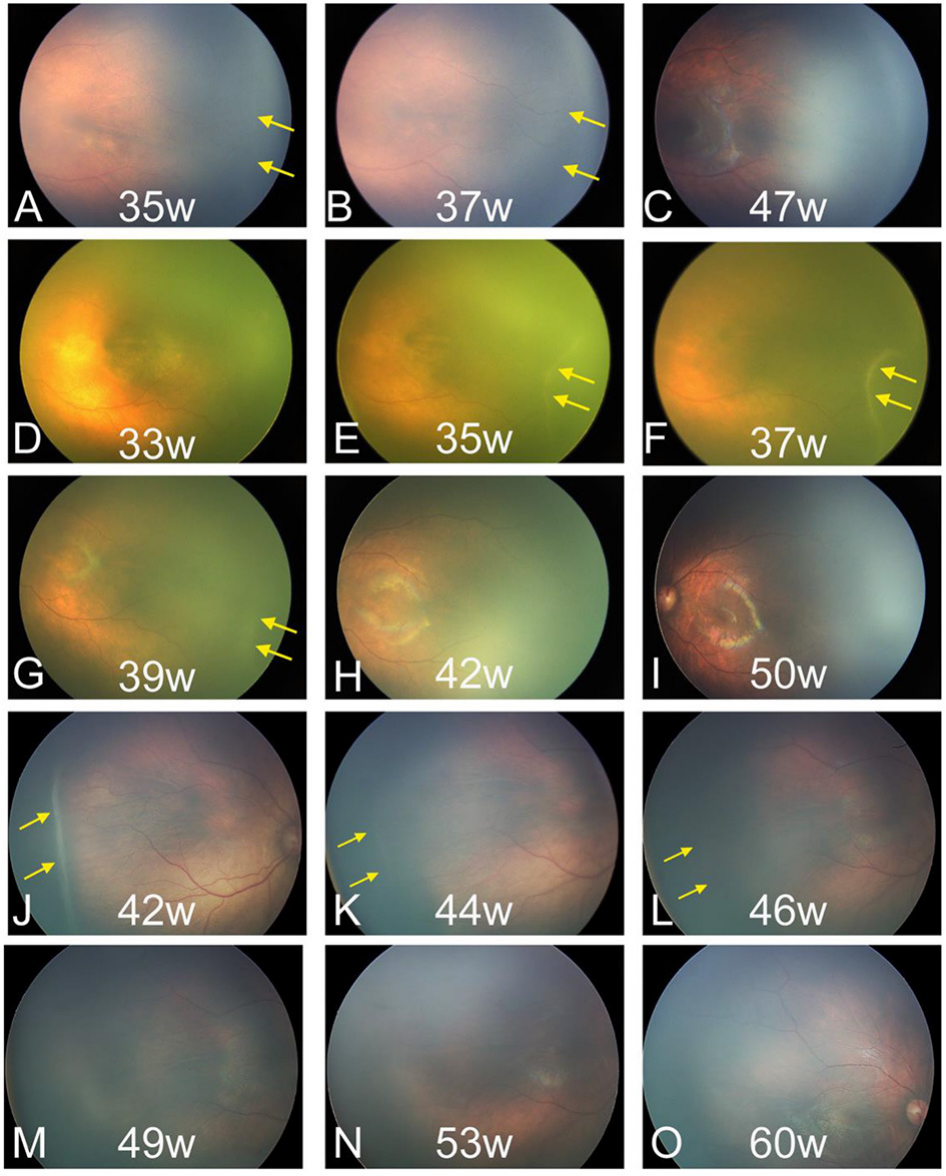
Fundus images of patients with spontaneous regression. **(A–C)** Regression of ROP in stage 1. **(D–I)** Regression of ROP in stage 2. **(J–O)** Regression of ROP in stage 3. ROP, retinopathy of prematurity. The yellow arrow indicates the location of the lesions.

All the 237 patients with ROP stage 1 to 3 showed spontaneous regression without treatment. During this process, the lesions gradually subsided, and the retinal vessels gradually vascularized completely. In this process of regression, the alleviation of tortuous and dilated retinal vessels may indicate early regression before the ridge acquires a flat appearance and subsides. In the final, 192 patients had complete vascularization. The fundus manifestations of regression changed across stages. The demarcation line subsided gradually until ROP resolution, and the vessels gradually grew into the avascular peripheral retina in stage 1 ROP (Figures 2A–C). ROP regression in stage 2 showed the typical characteristics of gradually flattened ridge and tortuous and dilated retinal vessels, which alleviated gradually (Figures 2D–I). Some small, isolated tufts (“popcorn”), which were present in a few cases, also disappeared. In stage 3 ROP, the neovascularization or extraretinal fibrovascular proliferation gradually subsided, and the ridge became flat and disappeared (Figures 2J–O).

**Figure 3:**
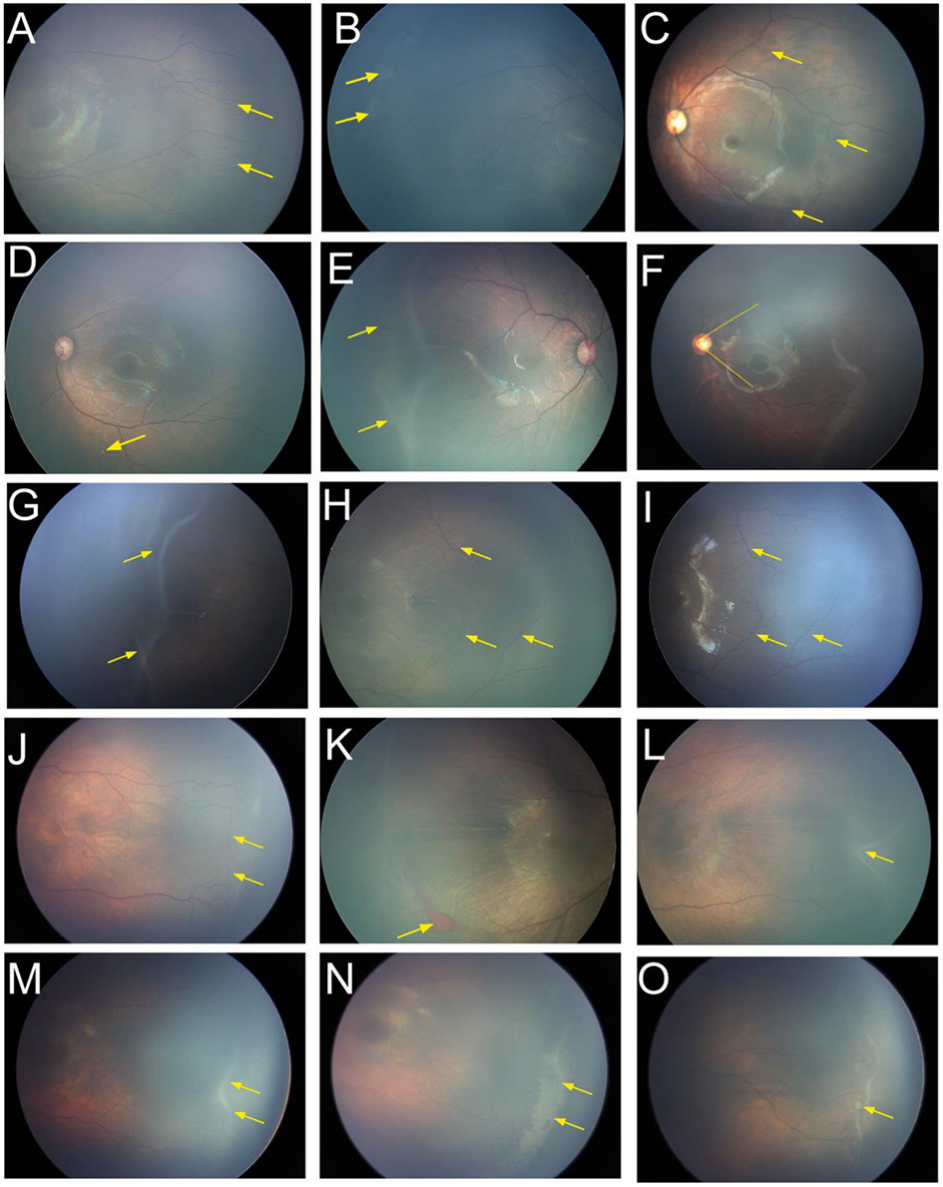
Fundus images of patients with spontaneous regression. **(A)** Increased vascular branching (yellow arrow) in the peripheral retina. **(B)** Gray flaky lesions (yellow arrow). **(C)** Retinal pigmentation and (yellow arrow). **(D)** Punctate retinal hypopigmentation (yellow arrow). **(E)** Ridge-like trace after the regression (yellow arrow). **(F)** Smaller acute angle between the upper and lower temporal retinal vessel trunks (yellow line). **(G)** Vitreous opacity or vitreous condensation (yellow arrow). **(H)** There were no tortuous or dilated retinal vessels (yellow arrow) during the acute phase at a postmenstrual age (PMA) of 41 weeks, **(I)** but the vessels became tortuous and dilated (yellow arrow) after ROP regressed at a PMA of 86 weeks. **(J)** Arteriovenous shunts and the formation of circumferential vessels (yellow arrow) were observed in the vascular–avascular junction. **(K)** Retinal hemorrhage (yellow arrow). **(L)** Irregular ridge (yellow arrow). **(M)** The ridge (yellow arrow) at a PMA of 40 weeks, **(N)** but it turned into a larger flattened patch (yellow arrow) at a PMA of 43 weeks and then disappeared gradually. **(O)** Popcorn (yellow arrow) was observed on the surface of the retina posterior to the ridge. ROP, retinopathy of prematurity; PMA, postmenstrual age.

Despite ROP regression, some fundus abnormalities persisted. Forty-five eyes (19.0%) did not show complete vascularization in the temporal retinal periphery. Sixteen patients (6.8%) showed increased vascular branching in the peripheral retina (Figure 3A). Gray, flaky lesions remained in 13 eyes (5.5%, Figure 3B). Thirteen eyes showed retinal pigmentary changes (5.5%); among them, seven eyes showed retinal pigmentation, and four eyes had punctate retinal hypopigmentation (Figures 3C,D). Nine patients (3.8%) showed ridge-like trace despite ROP regression (Figure 3E). A small acute angle between the upper and lower temporal retinal vessel trunks was found in seven eyes (3.0%, Figure 3F). Vitreous opacity or vitreous condensation occurred in six eyes (2.5%, Figure 3G).

Some special manifestations were observed during the regression. The retinal vessels in some infants did not return to normal despite complete ROP regression. Tortuous and dilated retinal vessels were detected in 25 infants (10.5%) at the beginning, and mildly tortuous or dilated vessels remained despite ROP regression in 15 cases (4.2%). Interestingly, we found no tortuous or dilated retinal vessels at the acute phase in six infants, but the vessels became tortuous and dilated after the ROP regressed (Figures 3H,I). Arteriovenous shunts and formation of circumferential vessels were observed in the vascular–avascular junction of 14 patients; they remained in three patients despite ROP regression (Figure 3J). RH was detected in 21 cases (8.9%), all of which were absorbed before the completion of regression (Figure 3K).

For infants with irregular ridge (13 cases), the ridge gradually became straight and then subsided, with complete regression at a median postmenstrual age (PMA) of 51.0 weeks (Figure 3L). The ridge in some infants did not decrease gradually during the course of regression. Instead, it turned into a larger flatten patch and then disappeared gradually over a longer period (Figures 3M,N). The popcorns were observed in seven infants on the retinal surface posterior to the ridge and subsided before the ridge regressed (Figure 3O). The regression completed in a median PMA of 56.3 weeks in these infants, longer than that for patients without popcorn.

### Time Course of Regression

**Figure 4:**
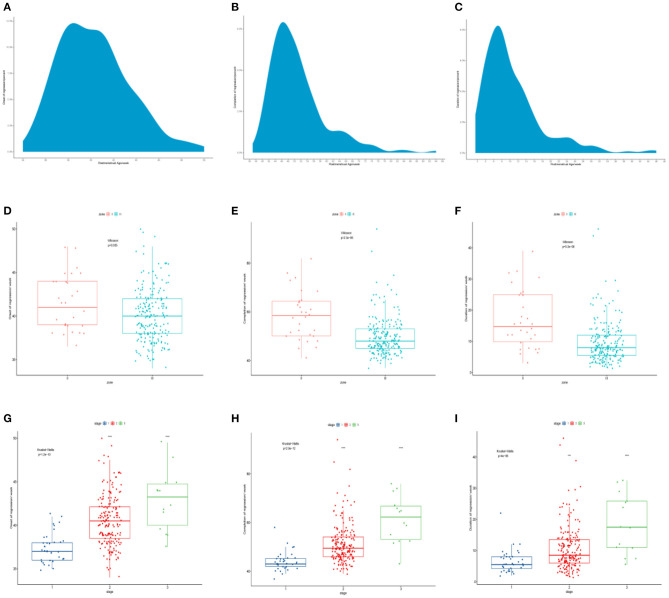
**(A–C)** The time course for regression onset, completion, and duration. **(D–F)** The comparison of time course of spontaneous regression of ROP among different zones. **(G–I)** The comparison of time course of spontaneous regression of ROP among different stages. ROP, retinopathy of prematurity.

The time for regression onset ranged between 36.0 and 46.0 weeks of PMA (5–95%), and the peak time ranged between 38.0 and 41.0 weeks, with a median PMA of 40.0 weeks. Furthermore, the time for regression completion varied (PMA range, 41.0–67.3 weeks; 5–95%), and the peak was between 46.0 and 50.0 weeks, with a median PMA of 49.0 weeks. The time for regression duration ranged from 3.0 to 25.1 weeks (5–95%), and the peak was between 6.0 and 10.0 weeks, with a median of 8.5 weeks (Figures 4A–C).

There were significant differences in the time course of spontaneous regression of ROP among different zones and stages (Table 2). The regression in zone III started at a median PMA of 40.0 weeks, earlier than in zone II (median PMA of 41.0 weeks; *p* = 0.035). ROP in zone III completed regression at a median PMA of 48.0 weeks, much earlier than that in zone II (median PMA of 58.5 weeks; *p* < 0.001). ROP in zone II required more time to complete the regression (median, 14.8 weeks) than ROP in zone III (median, 8.0 weeks; *p* < 0.001) (Figures 4D–F). In 237 patients, there was no ROP in zone I that regressed spontaneously.

**Table 2:** Time course of regression for ROP in different zones and stages.

	**No. of eyes**	**Onset of regression (median PMA, W)** **(5–95%)** ^*^	**Completion of regression (median PMA, W)** **(5–95%)** ^*^	**Duration of regression (median, W)** (5–95%)*
**Zone**				
II	30	41.0 (38.0–46.9)	58.5 (45.2–75.1)	14.8 (6.2–32.3)
III	207	40.0 (36.0–45.9)	48.0 (41.0–65.7)	8.0 (3.0–22.0)
*P-*value		0.035	<0.001	<0.001
**Stage**				
1	35	37.0 (35.4–40.7)	43.0 (39.7–50.5)	5.5 (2.9–12.0)
2	188	40.5 (36.5–46.0)	49.5 (42.2–67.0)	8.5 (3.0–24.7)
3	14	43.3 (38.5–48.5)	62.3 (48.6–74.7)	17.5 (6.8–32.2)
*P*-value		<0.001	<0.001	<0.001

*PMA, postmenstrual age; W, weeks*.

* *The range listed is the fifth to 95th percentile for this cohort*.

On the other hand, the analysis of regression time based on the stages showed that ROP stage 3 (median, 43.3 weeks) started regression later than ROP stage 2 (median, 40.5 weeks) and ROP stage 1 (median, 37.0 weeks) (*p* < 0.001). ROP stage 1 completed regression first (median, 40.5 weeks), while ROP stage 3 was the last to complete regression (median, 62.3 weeks) (*p* < 0.001). ROP stage 3 required more time to complete regression (median, 17.5 weeks) than ROP stage 2 (median, 8.5 weeks) and stage 1 (median, 5.5 weeks) (*p* < 0.001, in both cases) (Figures 4G–I).

### Factors Affecting Delayed Regression

**Figure 5:**
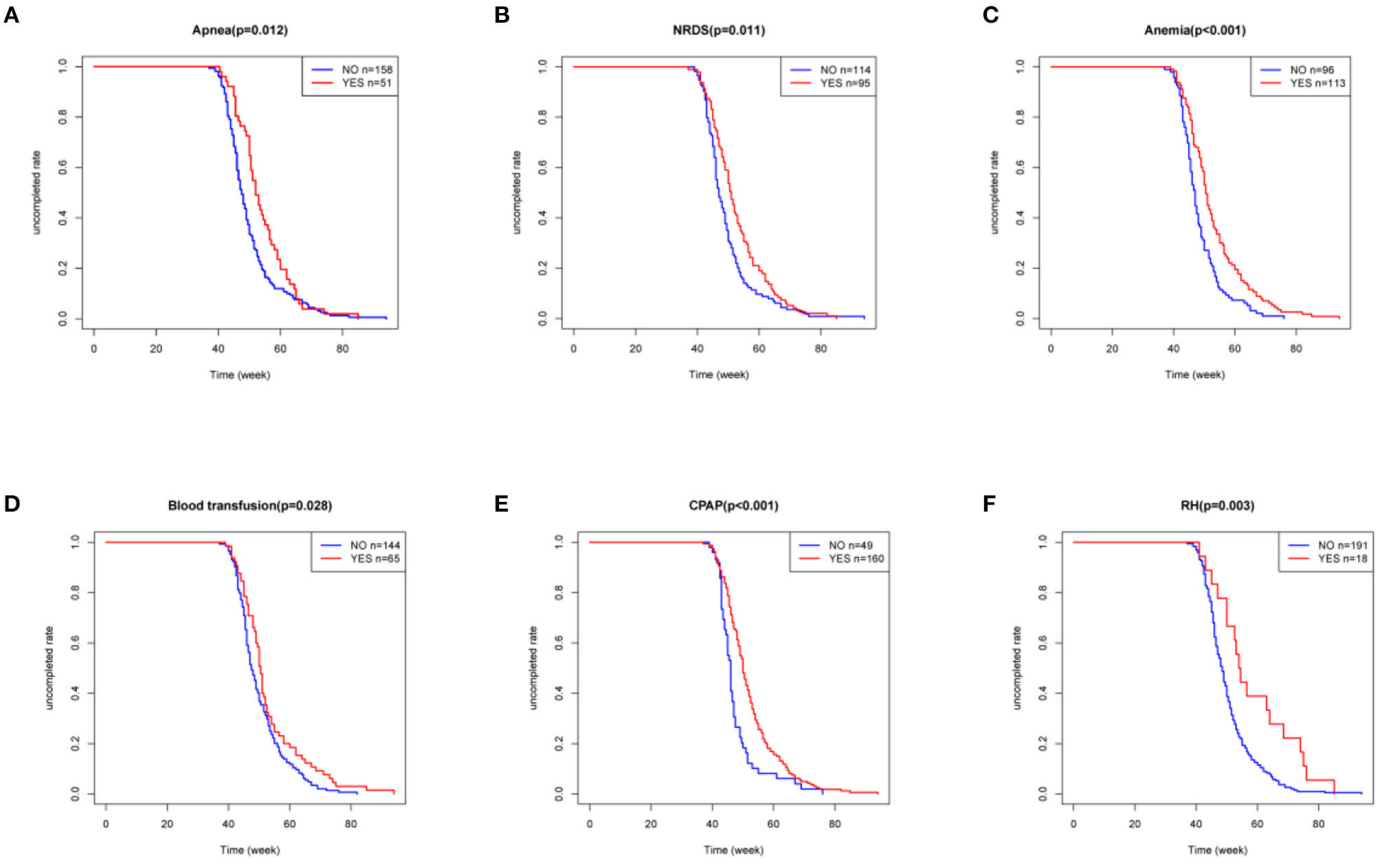
Kaplan–Meier curve was used to screen for factors related to delayed regression, and the differences were assessed by log-rank test. **(A–F)** These six factors were considered statistically significant, all *P* < 0.05. NRDS, neonatal respiratory distress syndrome; CPAP, continuous positive airway pressure; RH, retinal hemorrhage.

**Figure 6:**
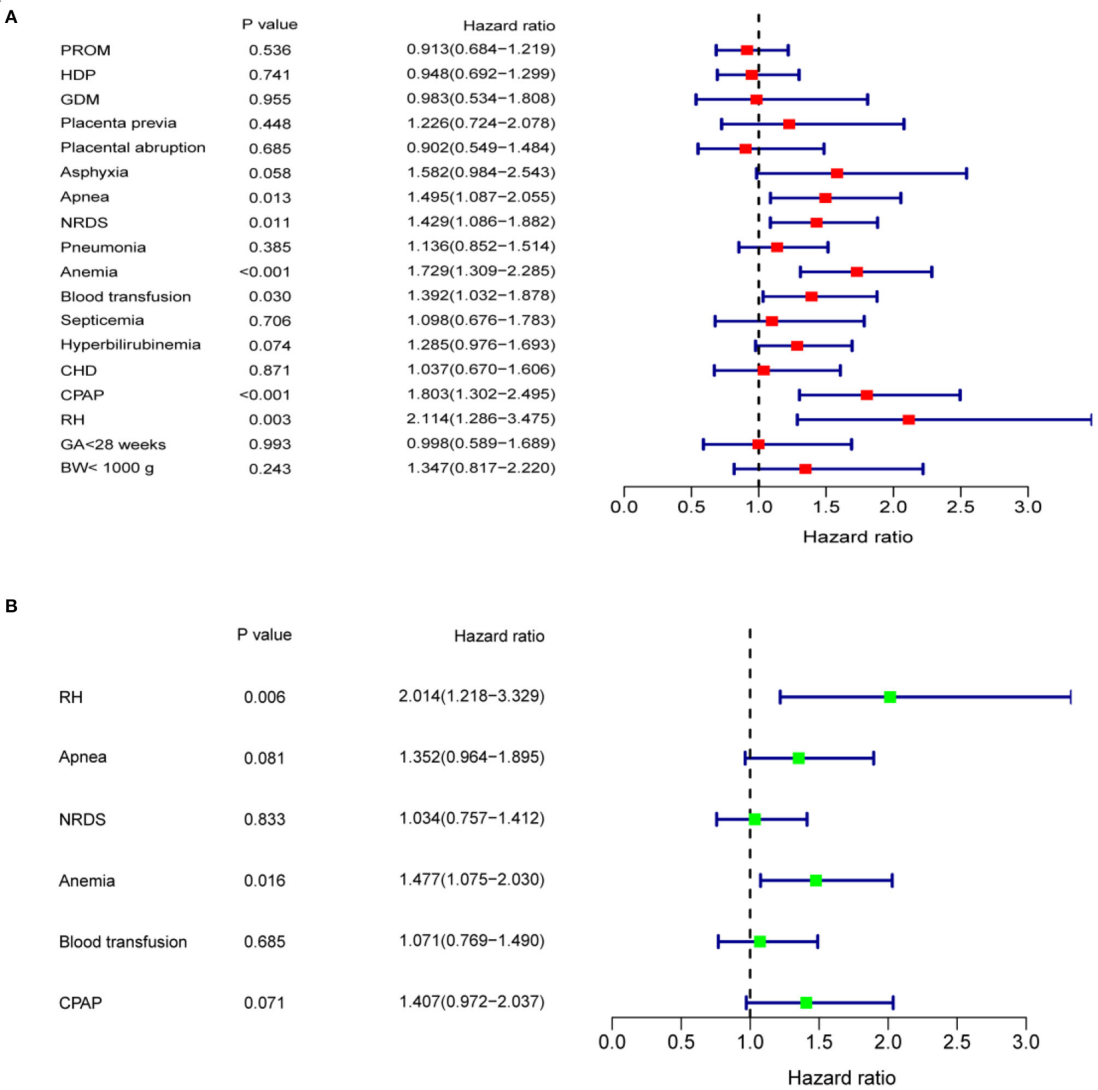
**(A)** Univariate Cox proportional hazard regression model showed that apnea, NRDS, anemia, blood transfusion, CPAP, and RH were closely associated with delayed regression. **(B)** Multivariate Cox proportional hazard regression model showed that anemia and RH were independent factors affecting delayed regression. BW, birth weight; CHD, congenital heart disease; CPAP, continuous positive airway pressure; GA, gestational age; GDM, gestational diabetes mellitus; HDP, hypertensive disorders of pregnancy; NRDS, neonatal respiratory distress syndrome; PROM, premature rupture of membranes; RH, retinal hemorrhage.

Since the ROP-associated factors in 28 infants were unclear, as they were not provided in the medical history or by their parents, only the data of 209 patients with ROP were finally included. In the univariate analyses, a Kaplan–Meier curve was used to screen for factors related to delayed regression, and the differences were assessed by log-rank test. The analysis showed that the patients with apnea, NRDS, anemia, blood transfusion, CPAP, and RH had longer time for completion of regression than those without these conditions (Figure 5, all *p* < 0.05). The univariate Cox proportional hazard regression model revealed that apnea (HR = 1.495, 95% CI = 1.087–2.055; *p* = 0.013), NRDS (HR = 1.429, 95% CI = 1.086–1.882; *p* = 0.011), anemia (HR = 1.729, 95% CI = 1.309–2.285; *p* < 0.001), blood transfusion (HR = 1.392, 95% CI = 1.032–1.878; *p* = 0.030), CPAP (HR = 1.803, 95% CI = 1.302–2.495; *p* < 0.001), and RH (HR = 2.114, 95% CI = 1.286–3.475; *p* = 0.003) were closely associated with delayed regression (Figure 6A). Multivariate Cox proportional hazard regression model confirmed that anemia (HR = 1.477, 95% CI = 1.075–2.030; *p* = 0.016) and RH (HR = 2.014, 95% CI = 1.218–3.329; *p* = 0.006) were independent factors for delayed regression (Figure 6B).

## Discussion

The number of infants requiring ROP screening has been increasing in China, which resulted in a prominent contradiction with the shortage of ROP-related medical resources. Excessive fundus examinations during ROP screening and follow-up increased not only the medical costs but also the risk of ocular and systemic adverse events (10). However, most patients with ROP regress spontaneously (11). Maly (12) reported that the incidence of spontaneous regression of ROP was 81.5%. In most cases, it usually occurs at stage 1, stage 2, or mild stage 3 (13). Eighty-five percent of infants with stage 1 ROP completed regression spontaneously, while the proportions of patients with stage 2 and 3 ROP who completed regression spontaneously are 56 and 25%, respectively (13).

ROP gradually disappeared without treatment or retained some traces of involution, achieving a relatively stable state defined as spontaneous regression of ROP (14, 15). It is characterized by lower stage and/or retinal vessel growth into a more peripheral area (9). Although the clinical manifestations of ROP have been clearly described, only few reports have assessed the changes in fundus appearance associated with spontaneous regression. We found that although most of the tortuous and dilated retinal vessels in regressed ROP were reduced, there were still mildly tortuous or dilated vessels in some patients despite ROP regression. These vascular abnormalities possibly occurred due to ROP itself (16, 17). It is worth noting that some vessels, which had not been abnormal previously, became tortuous and dilated eventually. This finding was not reported previously. The most common sequel of ROP regression is incomplete vascularization in the temporal peripheral retina (18). About 84.1% patients with avascular retina present peripheral findings in the future, such as lattice-like changes, retinal holes, and retinal tears (19). Approximately 3% patients in our cohort had popcorn on the retina, and recent research reported that popcorn was generally often indicative of the start of the regression of ROP (20). However, the results showed that regression of ROP with popcorn took longer than that without popcorn. The more acute angle between the upper and lower temporal retinal vessel trunks may be caused by the traction of fibrovascular proliferation. In addition, vitreous condensation and ridge-like traces may increase the risk of retinal holes and retinal detachment (20). Therefore, long-term follow-up of patients with spontaneous regression of ROP is needed.

Understanding the time course of spontaneous regression of ROP, especially the onset and completion of regression, is crucial to design efficient strategies for fundus screening. Although it does not eliminate the need for continuous screening of individual infants, it could provide guidance for doctors to carry out ROP screening and for national public health departments to estimate medical requirements (9). However, there is a lack of research on the time course of regression. Repka et al. (9) reported that the mean time of onset of regression was 38.6 weeks of PMA. In another study, without treatment, acute ROP started to regress at an average PMA of 40.4 weeks, with complete regression by 50.6 weeks (21). Herein, as the regression times were not normally distributed, we used the median as a measure of central tendency. Acute ROP started to regress at a median PMA of 40.0 weeks and completed at a median PMA of 49.0 weeks. The median time of duration time was 8.5 weeks. The time course in our study was similar to that reported previously (21). Importantly, our study had a larger sample size and more accurate description of the central tendency.

The time course for regression of different ROP zones and stages was significantly different, which was not quite the same with previous studies (21). The higher stages of ROP had a much later time of onset and completion and a longer duration. ROP in zone II started to regress later than ROP in zone III, and its regression took a longer time. However, a previous study reported no differences between different zones in the onset time of ROP (9, 21). Ninety-five percent of patients in our study started to regress before a PMA of 46 weeks, so the frequency of screening may be reduced after 46 weeks of PMA or after the onset of regression is identified. According to our results, the frequency of follow-up screening might also be reduced in patients who completed the regression in longer periods of time.

Despite numerous reports on the risk factors of ROP (10, 22–24), little is known about the factors affecting its spontaneous regression. Only two small-scale studies have identified the factors influencing ROP regression. Acute ROP in stage 3, anemia, and CPAP might be the factors affecting delayed regression, whereas RH showed an inverse weak correlation with spontaneous regression (11, 21). In our study, the higher stages of ROP tended to complete regression later, and ROP in zone II took longer time to complete regression than did zone III. Furthermore, we used the Kaplan–Meier curve to determine six factors for delayed regression, namely, apnea, NRDS, anemia, blood transfusion, CPAP, and RH. We used univariate and multivariate Cox proportional hazard regression models to analyze independent factors for delayed regression for the first time; and anemia and RH were confirmed as independent factors for delayed regression. Anemia has been regarded as a risk factor for severe ROP and ROP requiring treatment (25). Lower level of hemoglobin may decrease the oxygen-carrying capacity of tissues, and hypoxia might hinder the process of retinal vascularization and spontaneous regression (26, 27). RH was found to occur in 34% of neonates and to be more common in both eyes (28). It may be caused by abnormal retinal arteriovenous shunts and increased vascular fragility in patients with ROP (11). Therefore, it was speculated that RH indirectly reflects the severity of vascular abnormalities that might delay ROP regression.

This study has a few limitations. First, there may exist selection bias, as this was a single-center retrospective study. Our data are not representative of the entire population of China; although the patients came from a dozen provinces, they were mainly from the underdeveloped northwest regions of China. Further multicenter prospective studies are needed to verify our conclusion. Next, since regression of ROP is a continuous process, it is difficult to determine the exact time. The definition of regression time also affected the calculation of the time course. Then, it is clearer and more accurate to monitor the development of retinal vessels by fundus fluorescein angiography (FFA), while it is an invasive testing especially for premature children. For children with lighter ROP in our study, there were only 19 patients who accepted the FFA examinations. FFA should be used to further study the fundus performance, especially the changes of peripheral vascula in patients with the regression of ROP. Finally, further studies with longer follow-up periods are required for a more complete characterization of the abnormalities in the fundus, and to further understand visual development in these patients.

In conclusion, our study was a large cohort with complete long-term follow-up. We first systematically reported the changes of fundus appearance of spontaneous regression, especially some special manifestations during regression never reported before. Furthermore, we comprehensively described the time course of regression in infants with ROP in different zones and stages and found that ROP in zone II started to regress later than ROP in zone III for the first time. In addition, we found that RH is an independent factor affecting delayed regression for the first time and verified that anemia was also an independent factor. These findings will help understand more about the natural course of regression of ROP and may serve as a reference for developing more reasonable and effective screening and follow-up plans, thereby reducing frequency of the examination and the subsequent follow-up, saving unnecessary medical costs, and alleviating the contradiction between requirements of screening and lack of professionals, so as to promote the screening and prevention of ROP more efficiently.

## Data Availability Statement

The raw data supporting the conclusions of this article will be made available by the authors, without undue reservation.

## Ethics Statement

The studies involving human participants were reviewed and approved by the Ethics Committee of Xijing hospital. Written informed consent for participation was not provided by the participants' legal guardians/next of kin because: This study is a retrospective case series.

## Author Contributions

ML, ZZ, and YW: performing the screening and diagnosis of ROP. HY, LW, JF, YZ, and KG: collection and assembly of data. LW, JZ, and ZZ: data analysis and interpretation. All authors study conception and design, manuscript writing, and final approval of manuscript.

## Conflict of Interest

The authors declare that the research was conducted in the absence of any commercial or financial relationships that could be construed as a potential conflict of interest.

## Publisher's Note

All claims expressed in this article are solely those of the authors and do not necessarily represent those of their affiliated organizations, or those of the publisher, the editors and the reviewers. Any product that may be evaluated in this article, or claim that may be made by its manufacturer, is not guaranteed or endorsed by the publisher.

## References

[1] Hartnett ME Penn JS. Mechanisms and management of retinopathy of prematurity. N Engl J Med. (2012) 367:2515–26. 10.1056/NEJMra1208129 23268666 PMC3695731

[2] Gilbert C Rahi J Eckstein M O'Sullivan J Foster A. Retinopathy of prematurity in middle-income countries. Lancet. (1997) 350:12–4. 10.1016/S0140-6736(97)01107-0 9217713

[3] Limburg H Gilbert C Hon DN Dung NC Hoang TH. Prevalence and causes of blindness in children in Vietnam. Ophthalmology. (2012) 119:355–61. 10.1016/j.ophtha.2011.07.037 22035577

[4] Blencowe H Lawn JE Vazquez T Fielder A Gilbert C. Preterm-associated visual impairment and estimates of retinopathy of prematurity at regional and global levels for 2010. Pediatr Res. (2013) 74:35–49. 10.1038/pr.2013.205 24366462 PMC3873709

[5] Fierson WM. Screening examination of premature infants for retinopathy of prematurity. Pediatrics. (2018) 142:e20183061. 10.1542/peds.2018-3061 30478242

[6] Dou G Li M Zhang Z La Y Zhu Y Wang H. Demographic profile and ocular characteristics of stage 5 retinopathy of prematurity at a referral center in Northwest China: implications for implementation. BMC Ophthalmol. (2018) 18:307. 10.1186/s12886-018-0975-z 30497419 PMC6267009

[7] Li X. Screening guidelines of retinopathy of prematurity in China 2014. Chin J Ophthalmol. (2014) 50:933–5.

[8] International Committee for the Classification of Retinopathy of Prematurity. The International classification of retinopathy of prematurity revisited. Arch Ophthalmol. (2005) 123:991-9. 10.1001/archopht.123.7.991 16009843

[9] Repka MX Palmer EA Tung B. Involution of retinopathy of prematurity. cryotherapy for retinopathy of prematurity cooperative group. Arch Ophthalmol. (2000) 118:645–9. 10.1001/archopht.118.5.645 10815156

[10] Kim SJ Port AD Swan R Campbell JP Chan RVP Chiang MF. Retinopathy of prematurity: a review of risk factors and their clinical significance. Surv Ophthalmol. (2018) 63:618–37. 10.1016/j.survophthal.2018.04.002 29679617 PMC6089661

[11] Ju R-H Zhang J-Q Ke X-Y Lu X-Y Liang L-F Wang W-J. Spontaneous regression of retinopathy of prematurity: incidence and predictive factors. Int J Ophthalmol. (2013) 6:475–80. 10.3980/j.issn.2222-3959.2013.04.13 23991382 PMC3755307

[12] Maly E. Frequency and natural history of retinopathy of prematurity (Rop). A prospective study in a Swedish City 1986-1990. Acta Ophthalmol. (1993) 210:52–5. 10.1111/j.1755-3768.1993.tb04153.x 8329955

[13] Quinn GE Gilbert C Darlow BA Zin A. Retinopathy of prematurity: an epidemic in the making. Chin Med J. (2010) 123:2929–37. 21034609

[14] Prost M. [Possibilities of spontaneous regression in active phase of Rop]. Klin Oczna. (2003) 105:57–9. 12866174

[15] Fielder AR. The natural ocular outcome of premature birth and retinopathy: status at 1 year. Arch Ophthalmol. (1995) 113:850–1. 10.1001/archopht.1995.01100070020008 7605267

[16] Vural A Ekinci DY Onur IU Hergunsel GO Yigit FU. Comparison of fluorescein angiographic findings in type 1 and type 2 retinopathy of prematurity with intravitreal bevacizumab monotherapy and spontaneous regression. Int Ophthalmol. (2019) 39:2267–74. 10.1007/s10792-018-01064-7 30604251

[17] Mansukhani SA Hutchinson AK Neustein R Schertzer J Allen JC Hubbard GB. Fluorescein angiography in retinopathy of prematurity: comparison of infants treated with bevacizumab to those with spontaneous regression. Ophthalmol Retina. (2019) 3:436–43. 10.1016/j.oret.2019.01.016 31044736 PMC6501804

[18] Preslan MW Butler J. Regression pattern in retinopathy of prematurity. J Pediatr Ophthalmol Strabismus. (1994) 31:172–6. 10.3928/0191-3913-19940501-09 7931951

[19] Hamad AE Moinuddin O Blair MP Schechet SA Shapiro MJ Quiram PA. Late-onset retinal findings and complications in untreated retinopathy of prematurity. Ophthalmol Retina. (2020) 4:602–12. 10.1016/j.oret.2019.12.015 32059986 PMC7282927

[20] Xue K Huang X Xu S Zhnag T Wang X Zhnag M. The evolution of isolated neovascular tufts (“popcorn”) in retinopathy of prematurity. Retina. (2020) 40:1353–8. 10.1097/IAE.0000000000002596 31181037

[21] Ni Y-Q Huang X Xue K Yu J Ruan L Shan H-D. Natural involution of acute retinopathy of prematurity not requiring treatment: factors associated with the time course of involution. Invest Ophthalmol Vis Sci. (2014) 55:3165–70. 10.1167/iovs.13-13744 24764065

[22] Wade KC Ying G-S Baumritter A Gong A Kemper AR Quinn GE. Factors in premature infants associated with low risk of developing retinopathy of prematurity. JAMA Ophthalmol. (2019) 137:160. 10.1001/jamaophthalmol.2018.5520 30452500 PMC6439829

[23] Hellström A Smith LE Dammann O. Retinopathy of prematurity. Lancet. (2013) 382:1445–57. 10.1016/S0140-6736(13)60178-6 23782686 PMC4389630

[24] Yang Q Zhou X Ni Y Shan H Shi W Yin X. Optimised retinopathy of prematurity screening guideline in China based on a 5-year cohort study. Br J Ophthalmol. (2020) 105:819-23. 10.1136/bjophthalmol-2020-316401 32675062

[25] Lundgren P Athikarisamy SE Patole S Lam GC Smith LE Simmer K. Duration of anaemia during the first week of life is an independent risk factor for retinopathy of prematurity. Acta Paediatr. (2018) 107:759–66. 10.1111/apa.14187 29243312 PMC5902413

[26] Gariano RF Gardner TW. Retinal angiogenesis in development and disease. Nature. (2005) 438:960–6. 10.1038/nature04482 16355161

[27] Stutchfield CJ Jain A Odd D Williams C Markham R. Foetal haemoglobin, blood transfusion, and retinopathy of prematurity in very preterm infants: a pilot prospective cohort study. Eye. (2017) 31:1451–5. 10.1038/eye.2017.76 28548651 PMC5639193

[28] Emerson MV Pieramici DJ Stoessel KM Berreen JP Gariano RF. Incidence and rate of disappearance of retinal hemorrhage in newborns. Ophthalmology. (2001) 108:36–9. 10.1016/S0161-6420(00)00474-7 11150261

